# The Privy Council and Dr. Irvine

**Published:** 1901-06-01

**Authors:** 


### The Privy Council and Dr. Irvine.

An enthusiastic admirer of the Lord President of the Privy Council is reported to have said, many years ago, "What I like about Hartington is his you-be-damnedness;" and there can be no doubt that qualities which in the aggregate might be so described are not devoid of attractiveness in the eyes of the average Englishman. We question, nevertheless, whether the particular opportunity recently taken for the display of these qualities, in relation to the acts and proceedings of the General Medical Council, can be held to have been judiciously chosen. It appears that a pseudo-philanthropic "Institution" was formed at Birmingham, which had for one of its ostensible objects that of enabling working men to obtain the advice of eminent medical or surgical consultants at less than their customary fees. The consultants in question were invited to accept office under the Institution, and were then to be let out by its managers to such persons and on such terms as the managers might see fit. The net of this ingenious device having been vainly spread in the sight of the birds that it was intended to ensnare, the managers determined to appoint so-called "consultants" of their own selection, and to offer the services of these persons to the public by bold advertisement. One of those so appointed, or perhaps the only one, was a Dr. Irvine, who seems from the Medical Register to have obtained his first qualification as a Bachelor of Medicine of the Dublin University, in 1895, and to have proceeded to the Doctorate in 1897. According to a statement made by Sir John Gorst in the House of Commons, Dr. Irvine was "not a man of great means, and had supported himself, both before and after taking his degree by acting as a teacher and master in several secondary schools in Ireland." Dr. Irvine having accepted office under the "Institution," his supposed claims to public confidence as a "consultant" were advertised by the managers in terms which appeared, in the judgment of the Medical Defence Union, to exceed the limits of professional propriety, and he was accordingly charged with improper advertising, and was summoned before the Medical Council to answer to the charge. The Council, in hearing it, was acting in bare pursuance of its statutory duty, and it held that the fact of the advertising was proved. With regard to the character of the advertisements, it may be presumed that there was room for doubt whether they were such as to constitute, by mere acquiescence in their issue, infamous conduct in a professional respect; and the Council, as is their practice in such circumstances, adjourned the consideration of the charge until their next session, in order to give Dr. Irvine an opportunity of reconsidering his position, and of detaching himself, if he thought fit to do so, from the associations into which he had, perhaps unwittingly, fallen. He at once resigned his office as a "consultant" in the service of the "Institution;" and, before the arrival of the time at which, in the ordinary course, he could have cleared himself to the satisfaction of the Council, he was appointed by the Privy Council to be one of his Majesty's inspectors of schools. Dr. Irvine will, we have no doubt, make an excellent inspector; and, if his appointment had been delayed until the final judgment of the Medical Council had been declared, no criticism would have been called for. As matters stand, the appointment was grossly disrespectful to a legally constituted tribunal; and the disrespect was accentuated by the declaration of Mr. Chamberlain, in the House of Commons, that the only charge against Dr. Irvine was one of having infringed "the trades-union rule of the medical profession." The charge was one of complicity in the issue of misleading advertisements. But the real importance of the appointment is in the evidence it affords of position to which the Medical Council, in the estimation of the Government, and manifestly, also, in that of the House of Commons, has been reduced.

